# An Examination of Prospective Uses and Future Directions of Neuralink: The Brain-Machine Interface

**DOI:** 10.7759/cureus.14192

**Published:** 2021-03-30

**Authors:** Brian Fiani, Taylor Reardon, Benjamin Ayres, David Cline, Sarah R Sitto

**Affiliations:** 1 Neurosurgery, Desert Regional Medical Center, Palm Springs, USA; 2 Medicine, University of Pikeville-Kentucky College of Osteopathic Medicine, Pikeville, USA; 3 Medicine, Lyman Briggs College, Michigan State University, East Lansing, USA

**Keywords:** brain-machine interface, neuroprosthetics, neuronal amplification, artificial intelligence, neuralink, neuroengineering

## Abstract

The human brain is one of the most mystifying biological structures in nature. Overwhelming research, technology, and innovations in neuroscience have augmented clinical assessments, diagnosis, and treatment capabilities. Nonetheless, there is still much to be discovered about nervous system disorders and defects. Neuralink, a neurotechnology company, is advancing the field of neuroscience and neuroengineering. The company’s initial aim is to develop an implantable brain-machine interface device that will enhance the lives of people with severe brain and spinal cord injuries. Here, we provide insight into Neuralink’s design, early testing, and future applications in neurosurgery. While early testing with small and large animals show promising results, no clinical trials have been conducted to date. Additionally, a term search for “Neuralink” was performed in PubMed. The literature search yielded only 28 references, of which most indirectly mentioned the device but not in direct testing. In order to conclude the safety and viability of the Neuralink device, further research studies are needed to move forward beyond speculation.

## Introduction and background

Neuralink is a startup company by Tesla founder, Elon Musk, registered in 2016. Little information was known about the company until a white paper report was released on July 16, 2019 which detailed the purpose of the company and its various projects [1-3]. Neuralink as a company aims to “understand and treat brainly disorders,” “preserve and enhance our brain,” and “create a well-aligned future” [3].

Future goals of the company aim to help individuals with various forms of physical disability, enable humans to connect their brains to machines, and achieve symbiosis with artificial intelligence (AI) [2-4]. Some potential benefits within the medical community include helping individuals with controlling the movement of an exoskeleton or other robotics with their mind, allowing communication for patients with medical disabilities such as locked-in syndrome, restoring neuronal connections lost in degenerative disorders such as Alzheimer’s disease, monitoring and training of human’s psychophysiological states and cognitive abilities, and aiding in seizure prevention and treatment of drug-resistant epilepsy [1,2,5]. Outside of the medical community, Neuralink hopes to fill the void for when AI can simulate all our brainly functions and allow the human brain to compete with AI [3].

The idea behind Neuralink was to create a brain-machine interface (BMI) that could restore both sensory and motor function in individuals with neurological disorders [6]. The device utilizes “threads” which are tiny electrodes that can be surgically implanted into the brain of an individual using a robot designed by the Neuralink team. These electrodes provide real-time data on the activity of the selected group of neurons with more precision than electrodes used in deep brain stimulation (DBS) treatment for Parkinson’s disease. The data from the neurons is presented as “spike” patterns on the interface, similar to that of a depolarization current of an actual neuron. This data can be converted algorithmically and stored in the external Neuralink device, with the ability to personally observe this data via the Neuralink iPhone application. This technology has large implications in the field of neurosurgery. In this article, the authors aim to provide a brief background on the neurophysiology behind Neuralink, highlight the differences between Neuralink and DBS, highlight the technology behind the Neuralink system, and discuss its importance in future neurosurgical practice.

## Review

Neurobiology and technological inspiration behind Neuralink

The neuroscientific basis for the implementation of Neuralink stems from the standard physiology of neurons. When neurons receive enough depolarizing signals to exceed the depolarization threshold, they elicit action potentials that travel down their axons and cause the release of neurotransmitters that either potentiate or inhibit further signaling. Electrophysiological analysis of this process creates a “spike” pattern, which can be observed using probes and microelectrode arrays [7]. When a neuron is exposed to trauma, the efficacy of depolarization can be altered, either in frequency, amplitude, or both [8]. Restoration to other areas and structures of the body is far more efficacious than that of neurons. While there have been many in vitro studies showing neuronal regeneration, in vivo neuronal regeneration in the central nervous system has limited capacity and location, which are localized to the olfactory sensory system, dentate gyrus of the hippocampus, and the forebrain subventricular zone [9-11]. Even though restorative potential has been discovered, re-establishment of function is extremely limited when derived from these regions. Regeneration of function in neurons of the peripheral nervous system has increased potential as there are surgical interventions, such as autografts, that are used to improve functional outcomes. However, endogenous restoration of function after peripheral nerve transection is very rare and often not restored to initial functioning capacity [12]. A general assumption of poor endogenous restoration of both central and peripheral neurons, while understanding the physiology of why neurons work the way they do, provided motivation behind the creation of Neuralink.

Restoration of function using BMIs is a goal shared not only by Neuralink but technologies beforehand. Prior to the development of Neuralink, one of the mainstay therapies for movement disorders has been DBS [13]. While there are many mechanisms that contribute to DBS outcomes, their primary influences are on the electrical, neurochemical, and oscillatory actions of firing neurons [14]. High-frequency electrical stimulation is utilized to control the voltage-gated sodium channels within the neuronal tissue allowing regulation of action potential propagation [15,16]. Traditionally, DBS is believed to inhibit neural activity, but research has recorded increases in neural activity in the management of Parkinson’s disease demonstrating DBS effect to be tissue dependent [17].

Neuralink differs in two major ways from the traditional DBS approach: fiber design and electrode number. The Neuralink neuroprosthetic fiber design offers flexible, thin probes with an abundance of electrodes embedded into the fibers themselves creating increased biocompatibility in living tissue. Because of the flexible design, a robotic machine must be utilized to promote probe stiffness and ensure accurate placement throughout the brain. Rapid implantation of the Neuralink device includes placement of 96 probes, with each individual probe including 32 electrodes for a total of 3,072 electrodes placed throughout the brain [6]. Furthermore, the device is monitored by a Neuralink application-specific integrated circuit which provides concurrent tracing of the electrophysiologic data of the patient, unlike other conventional methods which track data offline. By doing this, Neuralink provides a 0.2 Hz greater threshold for true spike detection, maximizing the neuroprosthetic’s efficacy [6]. Neuralink seeks to build upon the foundation of DBS and provide hope for patients with poor outlooks on restoration of function.

Engineering innovation and early testing

The Neuralink device contains an array of 96 small, flexible electrode threads with 32 independent arrays per thread totaling 3,072 electrodes per array [3,18]. Previous research with DBS has allowed of control of computer cursors, robotic limbs, and speech synthesizers using no more than 256 electrodes [4].

The use of individually implanted threads with many electrodes allows for an exponential increase of channel count over older brain-machine technology [2,4]. This large number of electrodes allows for an increase in accuracy, classification, and interpretation of brain electrical activity and an increase in the transfer of a high volume of data which can be read, amplified, and sent to a machine for interpretation, or can be used to send signals to the brain to help in the treatment of other brain disorders [3,4,18]. Musk and his team have experimented with different metal and polymer types and believe a biocompatible polyimide over a gold, thin film trace is the best option for the threads. Each thread features an electrode contact and traces as well as a sensor area to communicate with the custom chip to enable signal amplification. The threads of an array are two layers of a conductor covered with three layers of insulation and measure 4-6 µm in diameter with a length of 20 mm ending in a 16 × 50 µm^2^ loop to aid in insertion [3]. Due to the extreme small nature of the threads, threads are placed on a parylene-c film until ready for insertion [3]. The use of flexible, polymer threads over traditional rigid metals allows for decreased immune response and better biocompatibility while allowing the threads to shift with brain movement and avoid brain vasculature [3].

The use of many flexible threads poses a surgical limitation during implantation. The smaller size and flexibility of the individual threads make it difficult to implant and create a very slow and tedious process. To overcome this obstacle, Neuralink has created a surgical robot capable of individually inserting each thread with high precision and safety while avoiding surface vasculature and targeting specific brain zones. This surgical robot accomplishes this task using image stacking from six different light modules capable of illuminating at 405 nm, 525 nm, and 650 nm and stereoscopic cameras. The different light illuminations allow for better visualization of the small thread and cortex of the brain and allow the robot to accurately light up and locate the thread loop for threading the needle and for precise insertion of the thread into the cortical surface. The robot uses preselected insertion sites, depth tracking, and landmarks on the skull to avoid brain vasculature efficiently and precisely during insertion. Testing of this robot over 19 rat models demonstrated an average of 87.1% insertion success rate [3].

Receiving and transcribing the information from over 3,000 electrodes proves another daunting task. The Neuralink device must be capable of recording, digitizing, and amplifying small neural signals (<10 µVRMS) while filtering out-of-band noise and streaming these signals out for real-time processing while still being compact and low power. The Neuralink device consists of 256 individually programmable amplifiers, on-chip-to-digital converters, and peripheral control circuitry for serializing the digitized outputs [3]. Neuralink uses a custom online spike-detection software to decode and display the signals. The signals are displayed on a graph with each row corresponding to an electrode site on the thread [3].

In an article published in 2019, Elon Musk detailed Neuralink’s two platforms planned to target the brain for neuroprosthetic applications [6,18]. The company designed two configurations, “System A” and “System B,” each differing in a number of variables. As described in the article, System A consisted of a 1,535 channel system with greater performance specifications than the 3,072 channel System B. Both systems were initially tested using male Long-Evans rats with unrestricted movement. Musk reported System A had the ability to record 1,344 out of the 1,535 channels simultaneously, while System B operated at a full 100% recording capacity. Furthermore, a threshold of >0.35 Hz was used to record spiking units. Utilizing System A, 40 of 44 device insertions were successful, yielding spikes in 43.4% of the channels. In other experiments, System A reported spike yields as high as 70% [6].

Future human trials will be critical for the exploration and validation of the neuroprostheses offered by Neuralink. While Neuralink aims for human trials to be completed in 2021, safety and biocompatibility of the human brain must be explored to validate the potential of the Neuralink device to adapt and control human neuronal impulses.

Unanswered questions and potential avenues

With an idea as radical as Neuralink, there are many questions surrounding not only its viability but its efficacy in clinical practice. Although implantation of the device requires both a Neuralink robot and live neurosurgeon, vast training is required by the neurosurgeon to increase comfortability and safety with the machine as the intention of the minute size of the electrode was to access anatomically protected neurons without harming adjacent vasculature. This required training with the device is a general contributing factor to the slow introduction of true robotic systems into neurosurgery and Neuralink is not excluded from this phenomenon, whereas co-robots, or “cobots,” are continuously implemented into neurosurgical practice [19].

The efficacy of this device is also limited by the specific types of pathologies that patients present with. Restoration of motor function in quadriplegic patients has been shown using a DEKA arm and BCI to translate neuronal activity into control signals to move the DEKA arm [20]. If the damage to the motor cortex or spinal cord is too severe, restoration of function by Neuralink or another BCI could prove challenging. Further development and interplay between BCI and in vivo neuronal regeneration are needed.

A potential direction of implementation of Neuralink into neurosurgical practice could be for classification and prevention of recurrence of brain tumors. A study performed by Hatcher et al. quantified peritumoral hyperexcitability of neurons surrounding glioblastoma multiforme (GBM) tumors in mice that led to recurrent seizures, a common presenting symptom in patients with GBM [18]. After surgical resection of the tumor, Neuralink threads could be placed at the tumor site and surrounding areas to detect potential increases in excitability of the neurons, indicating a need for further resection or enhanced chemotherapeutic treatments. This data could potentially reduce time of detection of recurrence and metastasis in patients with glioblastoma and other infiltrative cancers, as well as increase the life expectancy in these patients. While there are many uncertainties surrounding Neuralink, there is potential for implementation into future neurosurgical practice that could ultimately improve patient outcomes.

## Conclusions

The Neuralink implant and its potential for neuroamplification and treatment modalities give rise to hopeful advancements in improving the lives of people with spinal cord injuries, neurodegenerative disorders, and neurobiological shortfalls. However, although Neuralink’s technology and early testing outcomes appear promising, the requirement of neurosurgical robots to implant the device’s magnitude of electrodes raises safety and training concerns. To date, only speculations can be made regarding the safety and efficacy of the device. The paucity of data warrants further investigation and studies. Additionally, clinical trials are paramount for Neuralink to be accepted and integrated into the forefront of future neurosurgical practice.
